# Human apolipoprotein E promotes hepatitis B virus infection and production

**DOI:** 10.1371/journal.ppat.1007874

**Published:** 2019-08-08

**Authors:** Luhua Qiao, Guangxiang George Luo

**Affiliations:** Department of Microbiology, University of Alabama at Birmingham School of Medicine, Birmingham, Alabama, United States of America; The Pennsylvania State University College of Medicine, UNITED STATES

## Abstract

Hepatitis B virus (HBV) is a common cause of liver diseases, including chronic hepatitis, steatosis, fibrosis, cirrhosis, and hepatocellular carcinoma (HCC). HBV chronically infects about 240 million people worldwide, posing a major global health problem. The current standard antiviral therapy effectively inhibits HBV replication but does not eliminate the virus unlike direct-acting antivirals (DAA) for curing hepatitis C. Our previous studies have demonstrated that human apolipoprotein E (apoE) plays important roles in hepatitis C virus infection and morphogenesis. In the present study, we have found that apoE is also associated with HBV and is required for efficient HBV infection. An apoE-specific monoclonal antibody was able to capture HBV similar to anti-HBs. More importantly, apoE monoclonal antibody could effectively block HBV infection, resulting in a greater than 90% reduction of HBV infectivity. Likewise, silencing of apoE expression or knockout of apoE gene by CRISPR/Cas9 resulted in a greater than 90% reduction of HBV infection and more than 80% decrease of HBV production, which could be fully restored by ectopic apoE expression. However, apoE silencing or knockout did not significantly affect HBV DNA replication or the production of nonenveloped (naked) nucleocapsids. These findings demonstrate that human apoE promotes HBV infection and production. We speculate that apoE may also play a role in persistent HBV infection by evading host immune response similar to its role in the HCV life cycle and pathogenesis. Inhibitors interfering with apoE biogenesis, secretion, and/or binding to receptors may serve as antivirals for elimination of chronic HBV infection.

## Author summary

Little is known about the importance of host factors in HBV infection, assembly, and release due to the lack of robust cell culture models of HBV infection and propagation. The discovery of sodium taurocholate cotransporting polypeptide (NTCP) as the HBV receptor has made it possible to determine the roles of cellular genes in the modulation of HBV infection, assembly, maturation, and release. Through characterization of purified HBV, we found that human apoE is enriched in HBV and is incorporated onto the virus envelope, as demonstrated by immunoblot and immunoprecipitation using apoE-specific monoclonal antibodies and trypsin digestion. More importantly, HBV infection could be efficiently blocked by an apoE-specific monoclonal antibody or by silencing apoE expression and apoE gene knockout in the cell. These findings suggest that apoE likely mediates HBV cell attachment similar to its role in hepatitis C virus infection as previously demonstrated by us and others. Besides its importance in HBV infection, apoE is also required for efficient HBV production. Down-regulation of apoE expression or knockout of apoE gene from the HBV-producing hepatocytes severely impaired HBV production. Collectively, our findings demonstrate for the first time that apoE promotes both HBV infection and production.

## Introduction

Hepatitis B virus (HBV) infection continues to pose a major global health problem despite of the availability of effective HBV vaccine and antiviral drugs consisting of interferon (IFN) and nucleoside analogs (NAs). Currently, there are more than 240 million people chronically infected with HBV worldwide [1]. HBV vaccine has greatly reduced the number of new HBV infections and hepatocellular carcinoma (HCC) cases but does not offer therapeutic benefit to those chronically infected with HBV. Current antiviral regimens with NAs can effectively suppress HBV replication but are not curative unlike direct-acting antivirals (DAAs) for hepatitis C [2, 3]. Individuals with chronic HBV infection are at a substantial risk for progression to cirrhosis and HCC [4]. The World health organization has called for the elimination of viral hepatitis as a public health threat by 2030 [5].

The biggest challenge in eradicating chronic HBV infection is the elimination of its covalently closed circular DNA (cccDNA), which is the molecular basis for viral persistence [6, 7]. The current standard antiviral therapies are not sufficient for a complete or functional cure of chronic hepatitis B [2]. New classes of effective and safe antiviral drugs are urgently needed in order for the clearance of HBV. It is conceivable that effective antiviral therapy for curing chronic hepatitis B will likely require a combination of several drugs targeting different viral and/or cellular factors [6]. Thus, a more thorough understanding of HBV biology and the identification of novel targets are keys to the discovery and development of more effective anti-HBV drugs.

HBV belongs to the *Hepadnaviridae* family, a large group of small enveloped DNA viruses with a partially double-stranded DNA genome of about 3.2 kb [8]. Over the years, a great deal of new knowledge has been obtained about the underlying molecular mechanisms of HBV DNA replication [8], which undergoes reverse transcription of a pregenomic RNA (pgRNA) intermediate [9]. It encodes its own DNA polymerase that possesses both RNA-dependent DNA polymerase and RNase H activities, similar to reverse transcriptase of retroviruses. Additionally, the viral polymerase contains a terminal protein (TP) domain at its N-terminus that recognizes a unique RNA structure element designated epsilon (ε) located in the 5’-terminal repeat region of pgRNA and acts as a protein primer to initiate the viral minus-strand DNA synthesis [10]. Upon infection, the polymerase protein is removed from the relaxed circular DNA (rcDNA) genome in the cytoplasm, which is subsequently transported into the nucleus and converted to cccDNA [11, 12]. The rcDNA to cccDNA conversion is believed to be carried out by cellular enzymes, including DNA polymerase and ligase [13, 14], although the underlying molecular mechanism of cccDNA synthesis and maintenance remains unknown. The HBV cccDNA serves as a template for transcription of all viral mRNAs and pgRNA by the cellular Pol II polymerase. The viral mRNAs and pgRNA encode 7 proteins, three different forms (L, M, and S) of envelope proteins (HBs), preCore (HBe precursor), core (HBc), polymerase (P), and X protein (HBx) [8]. The pgRNA together with the attached viral polymerase is encapsidated by the core protein to form a nucleocapsid in which reverse transcription of the pgRNA takes place, resulting in the virion rcDNA genome [12]. The syntheses of viral RNA and DNA are modulated by many different cellular proteins [8]. In contrast to the advances made in HBV DNA replication, relatively little is known about cellular factors important for HBV infection and morphogenesis largely due to the lack of robust cell culture systems of HBV infection and propagation.

The discovery of sodium taurocholate cotransporting polypeptide (NTCP) as the HBV receptor has been a landmark advance in HBV research in recent years [15]. The expression of NTCP in nonpermissive human and murine hepatocytes conferred HBV susceptibility [15–24]. Recently, we have developed stable NTCP-expressing HepG2 (HepG2^NTCP^) and AML12 (immortalized mouse hepatocyte) cell lines that support robust HBV infection, replication, and morphogenesis in the presence of dimethylsulphoxide (DMSO) and hydrocortisone [25]. Using this newly developed HBV cell culture system and gene-specific small interfering RNAs (siRNAs), we have profiled a number of cellular genes in the modulation of HBV infection and morphogenesis. Interestingly, apoE-specific siRNAs were found to effectively reduce HBV infection similar to what we had previously observed for hepatitis C virus (HCV) [26].

Human apoE is a plasma exchangeable apolipoprotein associated with lipoproteins of various densities (VLDL, LDL, and HDL). It is a 34 kDa (299 amino acids) apoprotein containing a 22 kDa N-terminal domain (residues 1–191) that is recognized by receptors and a 10 kDa C-terminal domain (residues 222–299) that interacts with phospholipids [27–30]. There are three major apoE isoforms (apoE2, E3, and E4) in humans [31–33]. The N-terminal domain consists of four α-helix bundles with the receptor-binding site located in helix 4 [34]. ApoE is well known to play a central role in the transport, metabolism, and homeostasis of cholesterol and other lipids by serving as a ligand for low density lipoprotein receptors (LDLRs) and heparan sulfate proteoglycans (HSPGs). It is also involved in the repair response to tissue injury, cell growth and differentiation, immune regulation, and development of Alzheimer’s disease [35–37]. Interestingly, a number of independent studies have demonstrated that apoE is implicated in the life cycle and/or pathogenesis of different viruses [38], including but not limited to herpes simplex virus [39], human immunodeficiency virus [40], and HCV [26, 41, 42]. In the case of HCV, we have previously demonstrated that apoE is required for efficient HCV infection and production [26, 43]. Mechanistic studies revealed that apoE mediates HCV cell attachment by binding to HSPGs on the surface of hepatocytes and promotes HCV production through a specific interaction with HCV NS5A and E2 [41, 43–49].

The importance and underlying molecular mechanisms of apoE in the promotion of HBV assembly, maturation, or egress remain unknown. In the present study, we aimed to determine the association of apoE with HBV and its importance in the HBV life cycle in cell culture. Findings derived from our study demonstrate that apoE is enriched in purified HBV as determined by immunoblot and co-immunoprecipitation (co-IP) experiments using apoE-specific monoclonal antibodies. The apoE-blocking monoclonal antibody efficiently neutralized HBV infectivity. More importantly, silencing of apoE expression or knockout of apoE gene resulted in a reduction of greater than 90% of HBV infection and production. However, silencing or knockout of apoE gene did not affect HBV DNA replication or production of nonenveloped nucleocapsids. These novel findings demonstrate that apoE is required for efficient HBV infection and production.

## Results

### ApoE is enriched in purified HBV

**Fig 1:**
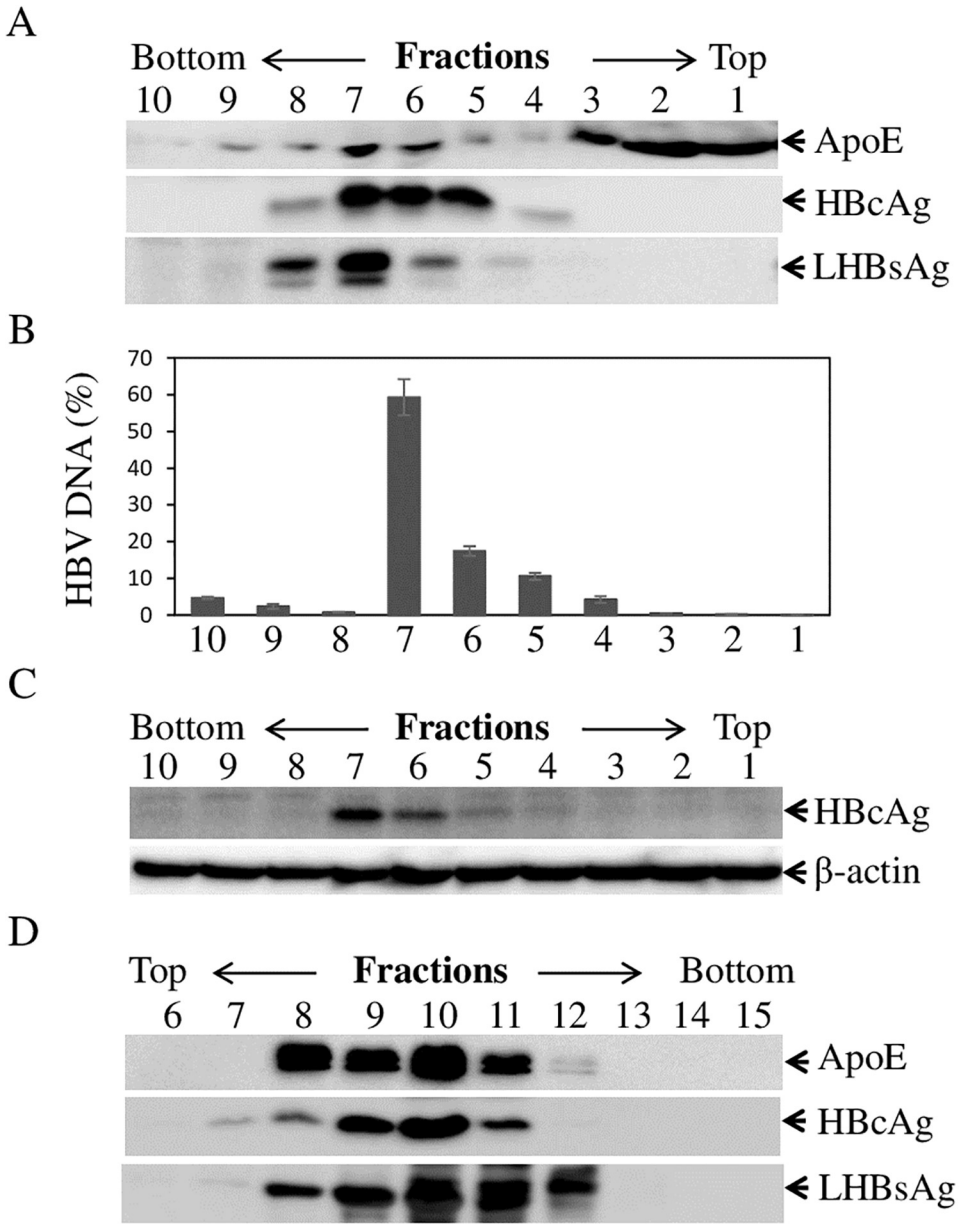
HBV purification and characterization. HBV produced from HepAD38 cells was concentrated by PEG 8000 precipitation and purified by CsCl gradient centrifugation and fractionation as described in materials and methods. Each fraction was used for the detection of apoE, HBc, and LHBsAg by WB (**A**) and HBV DNA by qPCR (**B**) as well as HBV infectivity (**C**). HBV was also precipitated using anti-HBs prior to CsCl gradient centrifugation and fractionation (D). For detection of apoE, HBcAg, and LHBsAg, individual fractions (numbered on the top) were subjected to electrophoresis in 10% SDS-PAGE. Upon transfer of proteins to PVDF membrane, apoE, HBcAg, and LHBsAg were stained with apoE-, HBc, and preS1-specific monoclonal antibodies. For quantification of HBV DNA, HBV DNA was extracted from 50 μl of each fraction and quantified by qPCR. HBV infectivity was determined by infecting HepG2^NTCP^ cells in 24-well cell culture plate with 25 μl of each fraction. At 4 days p.i., cell lysates were harvested for detection of HBc by WB using the monoclonal antibody T2221.

**Fig 2:**
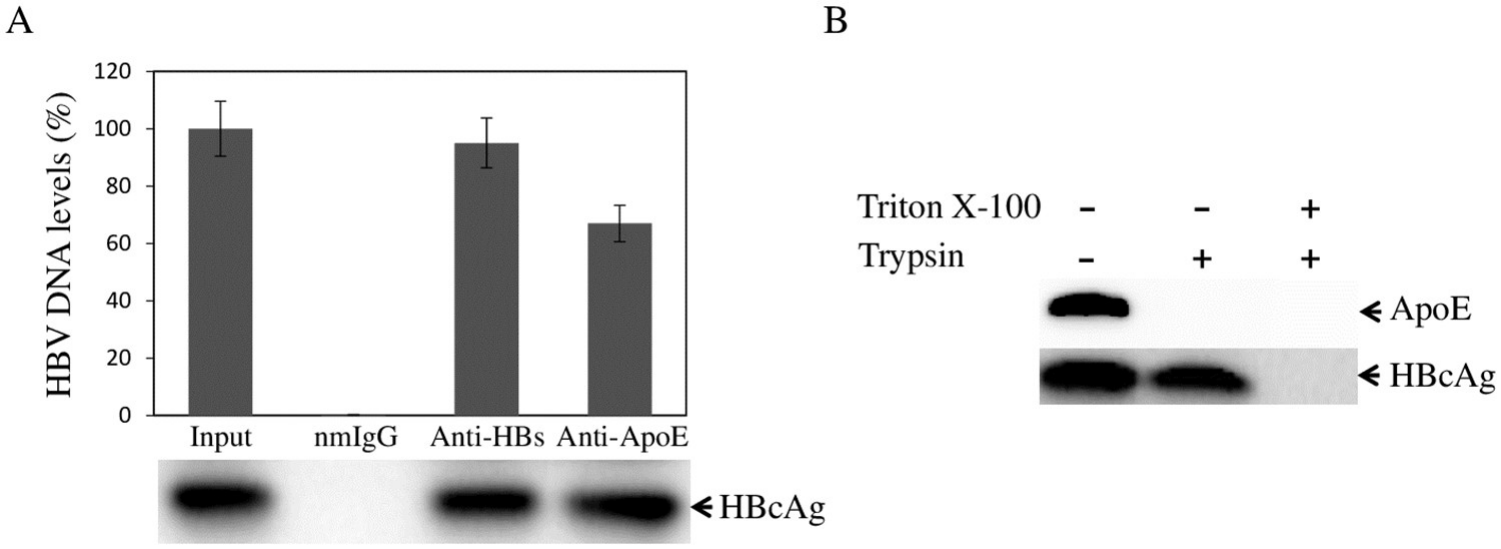
IP and trypsin digestion of purified HBV. **A**. Pull-down of purified HBV by HBIG and apoE-specific mAb23. Purified HBV in Fraction 7 (Fig 1) was incubated with HBIG- or apoE mAb23-conjugated beads. The normal mouse IgG (nmIgG)-conjugated beads were included as a control. Immunoprecipitated HBV was then used for extraction of HBV DNA, which was subsequently quantified by a qPCR method (graph), or detection of HBc by WB using the HBc-specific monoclonal antibody T2221 (bottom). The relative levels of HBV DNA pulled down by nmIgG, HBIG, or apoE mAb23 in triplicates are plotted considering the HBV DNA level of input HBV as 100%. **B**. Sensitivity of the HBV-associated apoE to trypsin digestion. Purified HBV was treated with 4 μg/mL of trypsin in the absence (-) or presence (+) of 1% Triton X-100 at 37°C for 1 h. HBc and apoE were detected by WB using HBc and apoE-specific monoclonal antibodies.

ApoE is predominantly produced by hepatocytes and is critical for lipid metabolism and homeostasis [35]. We and others had previously demonstrated that human apoE is incorporated into HCV particles and plays important roles in HCV infection and morphogenesis [26, 41, 43, 45–47, 49–53]. In present study, we sought to determine if apoE is also present in purified HBV. The cell culture grown HBV was concentrated with 10% PEG 8000 and was purified by cesium chloride gradient ultracentrifugation, followed by fractionation. The levels of apoE, HBcAg, LHBsAg, and HBV DNA in each fraction were determined by WB and qPCR, respectively. Additionally, the HBV infectivity of each fraction was determined by an HBV infection assay using HepG2^NTCP^-P3 cells, as described in our recent work [25]. The levels of HBcAg in the infected cells were subsequently determined by WB. As expected, most of apoE was detected in low-density fractions (1–3) containing the lipoproteins of various densities such as VLDL, LDL, and HDL (Fig 1A). Interestingly, significant levels of apoE were found in fractions 4 to 8 in which HBcAg, LHBsAg, and HBV DNA were also detected (Fig 1A and 1B). More importantly, the same fractions 4 to 8 contained infectious HBV with peak infectivity in fraction 7 (Fig 1C). To exclude possible contamination of HBV with lipoproteins, HBV virions and subviral particles were pulled down by IP using anti-HBs monoclonal antibodies prior to gradient ultracentrifugation and fractionation. Again, apoE was detected in the affinity-purified HBV as determined by presence of both HBcAg and LHBsAg (Fig 1D), demonstrating a *bona fide* association of apoE with HBV. There might be preS1-containing subviral particles in fractions 11 and 12 based on the higher ratios between LHBsAg and HBcAg than those in fractions 8 to 10. ApoE was also detected in fractions 11 and 12 although at lower levels (Fig 1D). Thus, it is still not clear if apoE interacts with HBV subviral particles. To further confirm the presence of apoE in purified HBV, fraction 7 with the highest level of infectious HBV was subjected to IP using HBsAg- and apoE-specific antibodies as well as a normal mouse IgG (nmIgG) as a control. The levels of HBV DNA extracted from the precipitated HBV were quantified by qPCR, while HBcAg in the precipitated HBV was detected by WB. Anti-HBs antibody pulled down greater than 95% of HBV, whereas the apoE mAb23 precipitated about 70% of HBV particles. However, similar levels of HBcAg were detected by WB between anti-HBs- and apoE mAb23-precipitated HBV (bottom, Fig 2A). In contrast, normal mouse IgG failed to pull down any detectable HBV (Fig 2A). These results suggest that apoE is incorporated onto HBV envelope. To further validate apoE on the HBV envelope, a trypsin digestion experiment was performed. Purified HBV in fraction 7 was treated with trypsin in the absence or presence of an envelope-disrupting detergent Triton X-100. ApoE was completely digested by trypsin in the absence of Triton X-100. The HBV capsids (HBcAg) were digested only after the viral envelope was disrupted by treatment with Triton X-100. These findings suggest that apoE is on the HBV envelope and is likely involved in HBV infection.

### Neutralization of HBV infectivity by an apoE-specific monoclonal antibody

**Fig 3:**
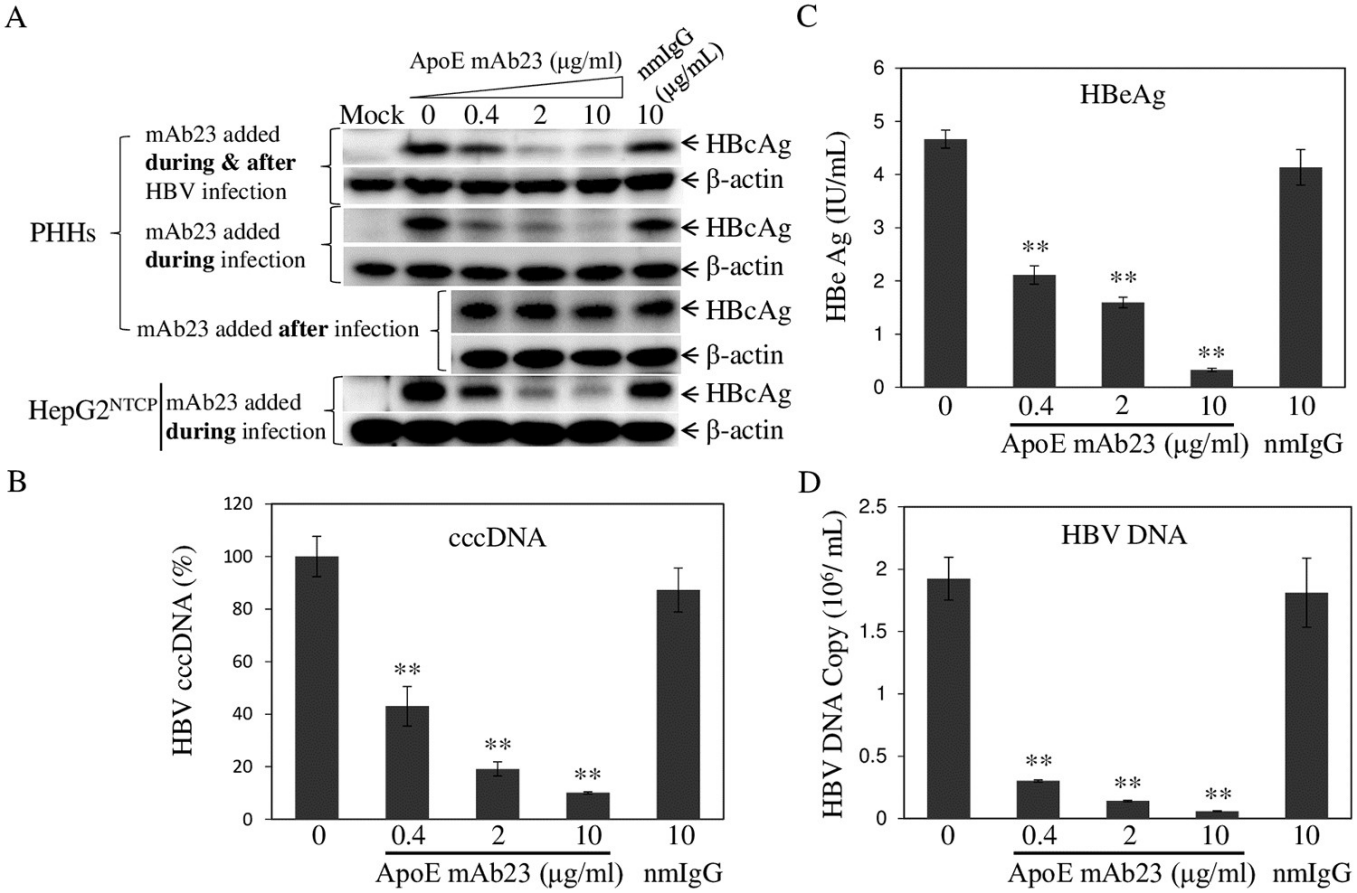
Neutralization of HBV infectivity by apoE mAb23. PHHs or HepG2^NTCP^ cells were seeded in 24-well cell culture plates at one day prior to HBV infection. PHHs or HepG2^NTCP^ cells were infected with HBV in the presence of 4% PEG 8000. For HBV neutralization, varying concentrations (0, 0.4, 2, and 10 μg/mL) of the apoE mAb23 or 10 μg/mL of nmIgG were added during HBV infection at 37°C for 12 h. HBV-infected PHHs or HepG2^NTCP^ cells were maintained in Power primary HEP medium with (mAb23 added during & after HBV infection) or without (mAb23 added during infection) the addition of corresponding concentrations of mAb23 or nmIgG for 4 days. PHHs were also infected with HBV in the absence of mAb23, which was added to cell culture media only after HBV infection (mAb23 added after infection). Cell lysates were then used for detection of HBcAg by WB with β-actin as an internal control (**A**). Additionally, HBV-infected PHHs with mAb23 added during and after infection were used for extraction of HBV cccDNA, which were quantified by a cccDNA-specific qPCR method (**B**). The levels of HBeAg in the cell culture supernatants were quantified by a chemiluminescence immunoassay (**C**). HBV DNA in the cell culture supernatants was extracted with a QIAGEN kit and was quantified by qPCR (**D**). The graphs represent average values of triplicates. ** P<0.01.

To determine if HBV-associated apoE is required for HBV infection, we carried out HBV neutralization experiments in PHHs and HepG2^NTCP^ using an apoE-specific monoclonal antibody (mAb23), which was previously shown to potently block HCV infection [26, 46]. PHH and HepG2^NTCP^ were infected with HBV in the presence of 4% PEG. Varying concentrations (0, 0.4, 2, and 10 μg/ml) of apoE mAb23 were added during HBV infection, during and after HBV infection, or only after HBV infection, as indicated on the left of Fig 3A. A normal mouse IgG (nmIgG) was used as a negative control. The levels of HBcAg and HBV cccDNA in the HBV-infected cells and the levels of HBeAg and HBV DNA in the cell culture supernatants were quantified by WB, chemiluminescence immunoassay, and qPCR, respectively. Strikingly, apoE mAb23 added during and after HBV infection effectively neutralized HBV infectivity in a dose-dependent manner, resulting in 84% reduction of HBcAg (Fig 3A top) and 90% decrease of HBV cccDNA (Fig 3B) in the cell and greater than 90% lower levels of HBeAg (Fig 3C) and HBV DNA (Fig 3D) in the cell culture supernatants at concentrations up to 10 μg/ml. More significantly, apoE mAb23 neutralized HBV infectivity when added only during HBV infection as efficiently as its presence during and after HBV infection (Fig 3A). However, apoE mAb23 did not affect the levels of HBcAg expression when added after HBV infection. HBV infection of HepG2^NTCP^ cells was also efficiently blocked by apoE mAb23 when added during HBV infection (Fig 3A bottom). These findings demonstrate that HBV-associated apoE does play an important role in HBV infection.

### ApoE expression on target cells is required for efficient HBV infection

**Fig 4:**
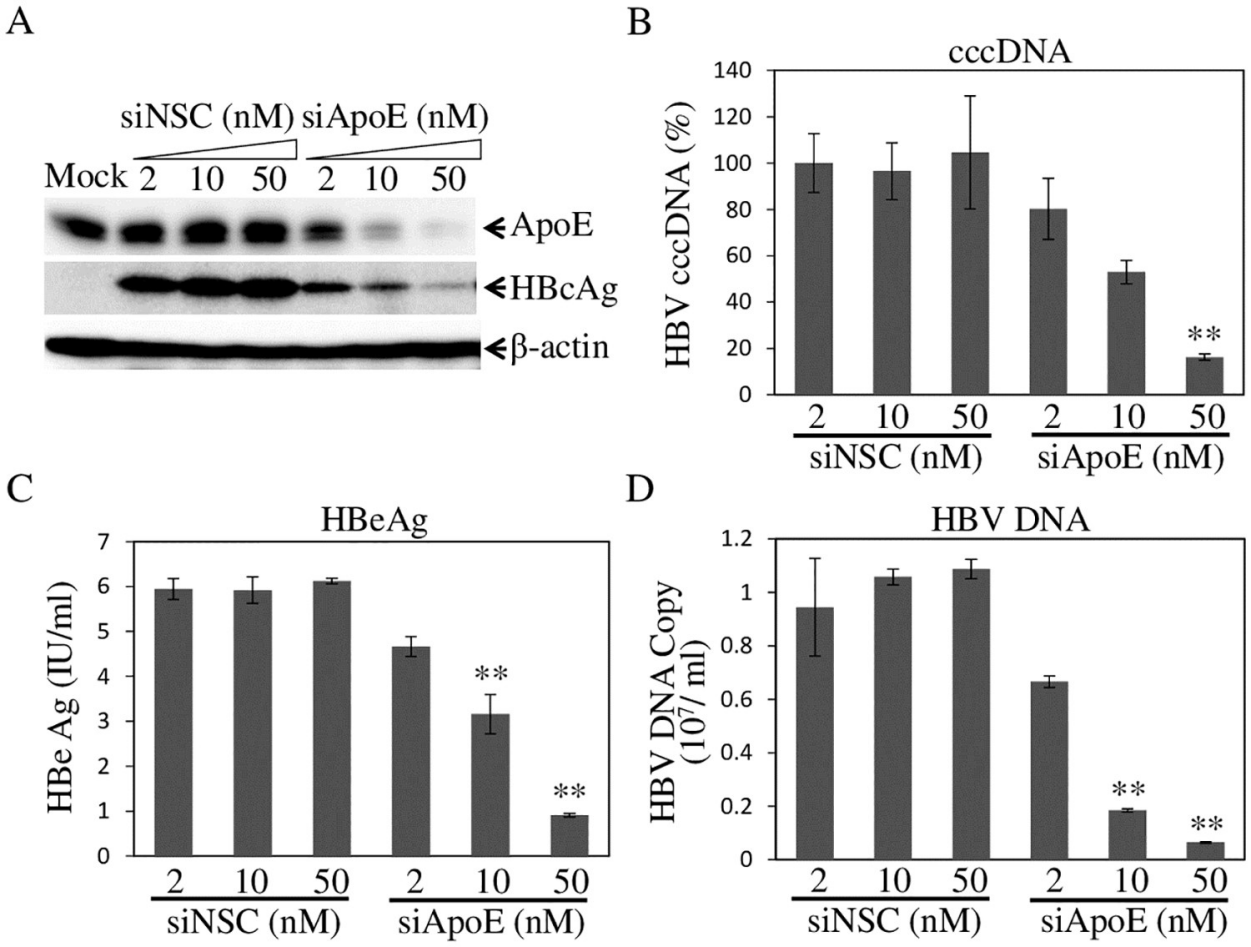
Effects of the siRNA-mediated knockdown of apoE expression on HBV infection. HepG2^NTCP^ cells were transfected with apoE-specific siRNAs (siApoE) at varying concentrations (0, 2, 10, 50 nM). An NSC siRNA (siNSC) was used as a negative control. At 48 h p.t., cells were infected with HBV for 12 hours. After 4 days p.i., the levels of apoE and HBc were determined by WB (**A**). HBV cccDNA in the cell was extracted with the Hirt method and quantified by qPCR (**B**). The levels of HBeAg in the cell culture supernatants were quantified by chemiluminescence immunoassay (**C**). HBV DNA in the cell culture supernatants was extracted with a QIAGEN DNA isolation kit and quantified by qPCR (**D**). Average values of triplicates are plotted in B, C, and D. ** P<0.01.

**Fig 5:**
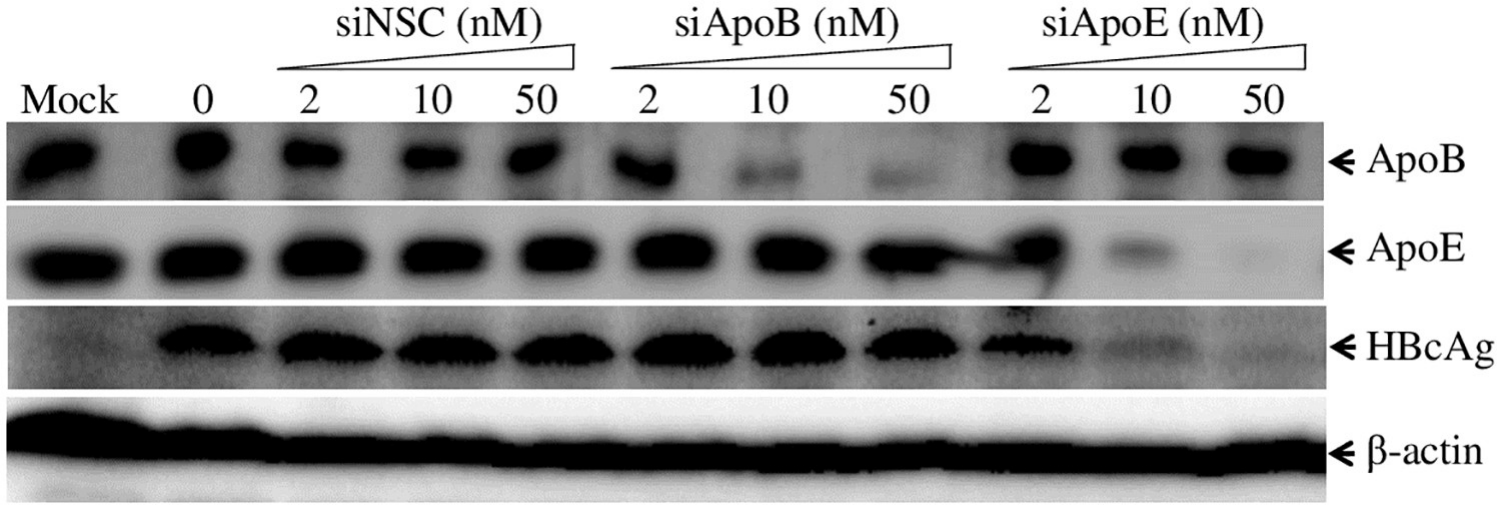
Reduction of HBV infection by silencing apoE but not apoB expression. HepG2^NTCP^ cells in 24-well cell culture plates were transfected with NSC-, apoB-, or apoE-specific siRNAs at varying concentrations (0, 2, 10, 50 nM). At 48 h p.t., cells were infected with HBV for 12 hours. After 4-days incubation with DME/F12 medium containing 4% FBS, 1% DMSO, and 5μg/mL hydrocortisone, the levels of apoB and apoE in the cell culture supernatants and HBc in the cell were determined by WB using apoB-, apoE-, and HBc-specific monoclonal antibodies.

**Fig 6:**
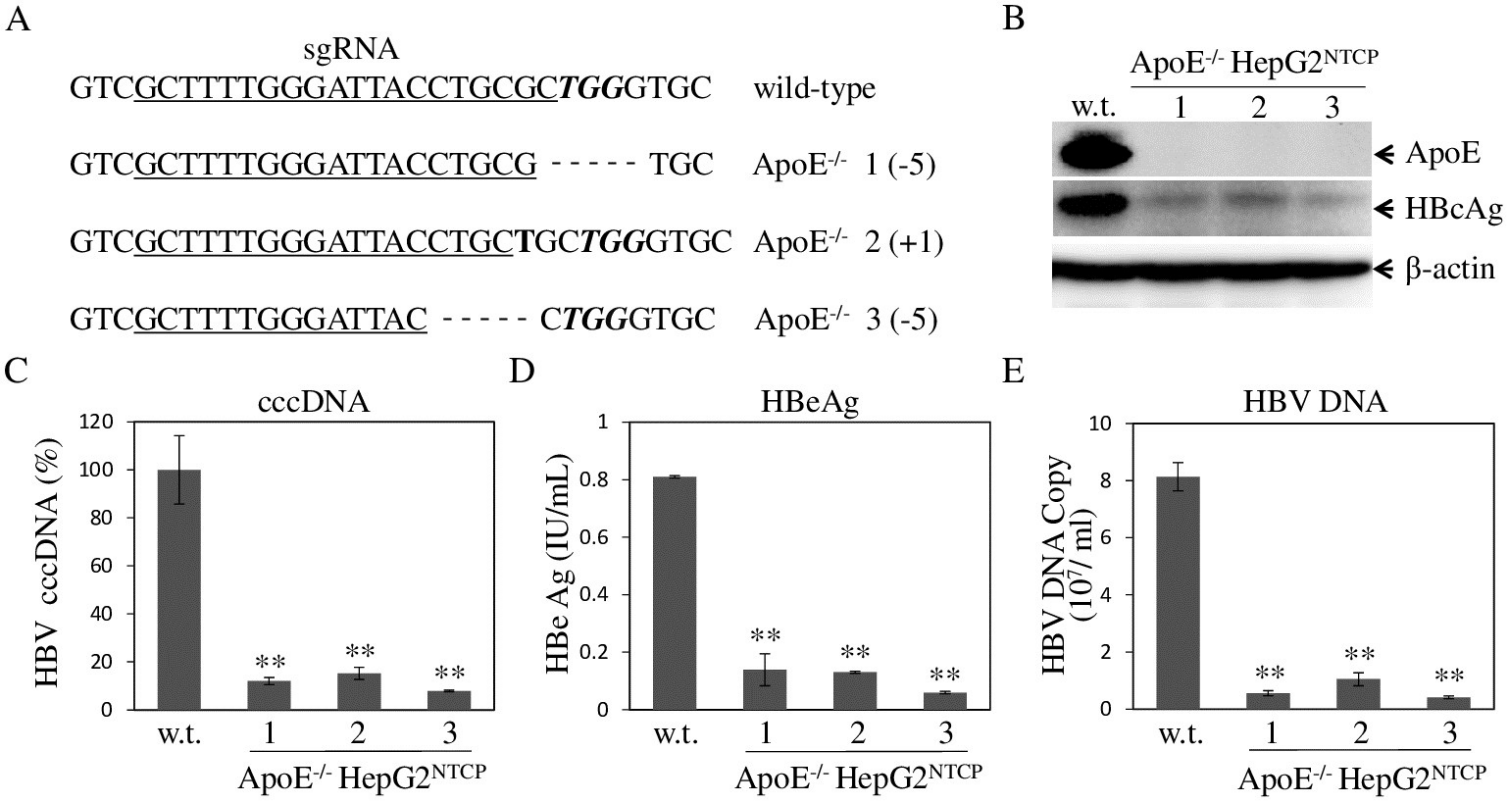
Impairment of HBV infection by apoE gene knockout. HepG2^NTCP^ cells were transduced with a lentivirus expressing CRISPR/Cas9 and apoE-specific sgRNA. Upon selection with blasticidin, stable cell clones were picked up and expanded. Genomic DNA was extracted with a QIAGEN DNA isolation kit. ApoE DNA fragments were amplified by PCR and indels were determined by DNA sequence analysis. Three apoE knockout (apoE^-/-^) cell lines contained 5 nucleotide deletion (-5) or one nucleotide insertion (+1) (**A**). The parental and apoE^-/-^ HepG2^NTCP^ cells in 24-well cell culture plates were infected with HBV. At 4 days p.i., the levels of apoE and HBc in the cell were determined by WB (**B**). HBV cccDNA in the cell was extracted with the Hirt method and quantified by qPCR (**C**). The levels of HBeAg in the cell culture supernatants were quantified by chemiluminescence immunoassay (**D**). HBV DNA in the cell culture supernatants was extracted with a QIAGEN DNA isolation kit and quantified by qPCR (**E**). Average values from three repeats were calculated for plotting in C, D, and E. ** P<0.01.

The studies by others demonstrated that apoE expression on target cells was important for HCV infection [54, 55]. The question arose whether apoE expression on target cells is also required for HBV infection. Initially, apoE expression in HepG2^NTCP^ cells was silenced by specific siRNAs prior to HBV infection. A non-specific control (NSC) siRNA was used as a control. At 48 hours after siRNA transfection, HepG2^NTCP^ cells were infected with HBV. At 4-d p.i., the levels of apoE expression, HBcAg, and HBV cccDNA in the cell as well as HBV DNA in the supernatants were determined by WB and qPCR, respectively. As expected, apoE-specific siRNAs efficiently silenced apoE expression in a dose-dependent fashion, resulting in a reduction of apoE by 40%, 64%, and 78% at 2, 10, and 50 nM concentrations. In contrast, apoE expression was not affected by NSC siRNA (Fig 4A). As a result, the levels of HBcAg (Fig 4A) and HBV cccDNA (Fig 4B) in the HBV-infected cells were all proportionally decreased by increasing concentrations of apoE siRNAs. The levels of HBV cccDNA were lowered by 20%, 47%, and 84% at 2, 10, and 50 nM of apoE siRNAs (Fig 4B). Similarly, the levels of HBeAg and HBV DNA in the cell culture supernatants were decreased by greater than 90% at 50 nM of apoE-specific siRNAs (Fig 4C and 4D). There is a close correlation between the reduction of apoE expression and the decrease of HBV infection, as determined by the levels of HBcAg, HBV cccDNA, HBeAg, and HBV DNA in the infected cells and supernatants (Fig 4). To determine the specificity of apoE in HBV infection, apoB was chosen as another control as we previously found that apoB was not required for HCV infection [46]. As expected, both apoB- and apoE-specific siRNAs effectively silenced their corresponding expression. However, only apoE but not apoB siRNAs greatly reduced HBV infection (Fig 5), demonstrating a specific requirement of apoE for HBV infection. To further validate the importance of apoE in HBV infection, we have also made stable apoE gene-knockout (apoE^-/-^) HepG2^NTCP^ cell lines. As shown in Fig 6A, three apoE^-/-^ cell lines contain either 5-nucleotide (nt) deletion (-5) or 1-nt insertion (+1) in the apoE open-reading frame (ORF). The lack of apoE expression in knockout cell lines was confirmed by WB (Fig 6B). The effects of apoE gene knockout on HBV infection were determined by HBV infection of these apoE^-/-^ HepG2^NTCP^ cell lines. Strikingly, apoE knockout resulted in 4-, 5-, and 7-folds reduction of HBcAg, respectively, in the HBV-infected cell lines (Fig 6B). Similarly, the levels of HBV cccDNA in the HBV-infected apoE^-/-^ cells were 90% lower than those in the parental HepG2^NTCP^ cells (Fig 6C). The levels of HBeAg (Fig 6D) and HBV DNA (Fig 6E) in the cell culture supernatants were reduced by greater than 90%. Collectively, these findings demonstrate that apoE expression on target cells is critical for efficient HBV infection.

### ApoE is important for HBV production

**Fig 7:**
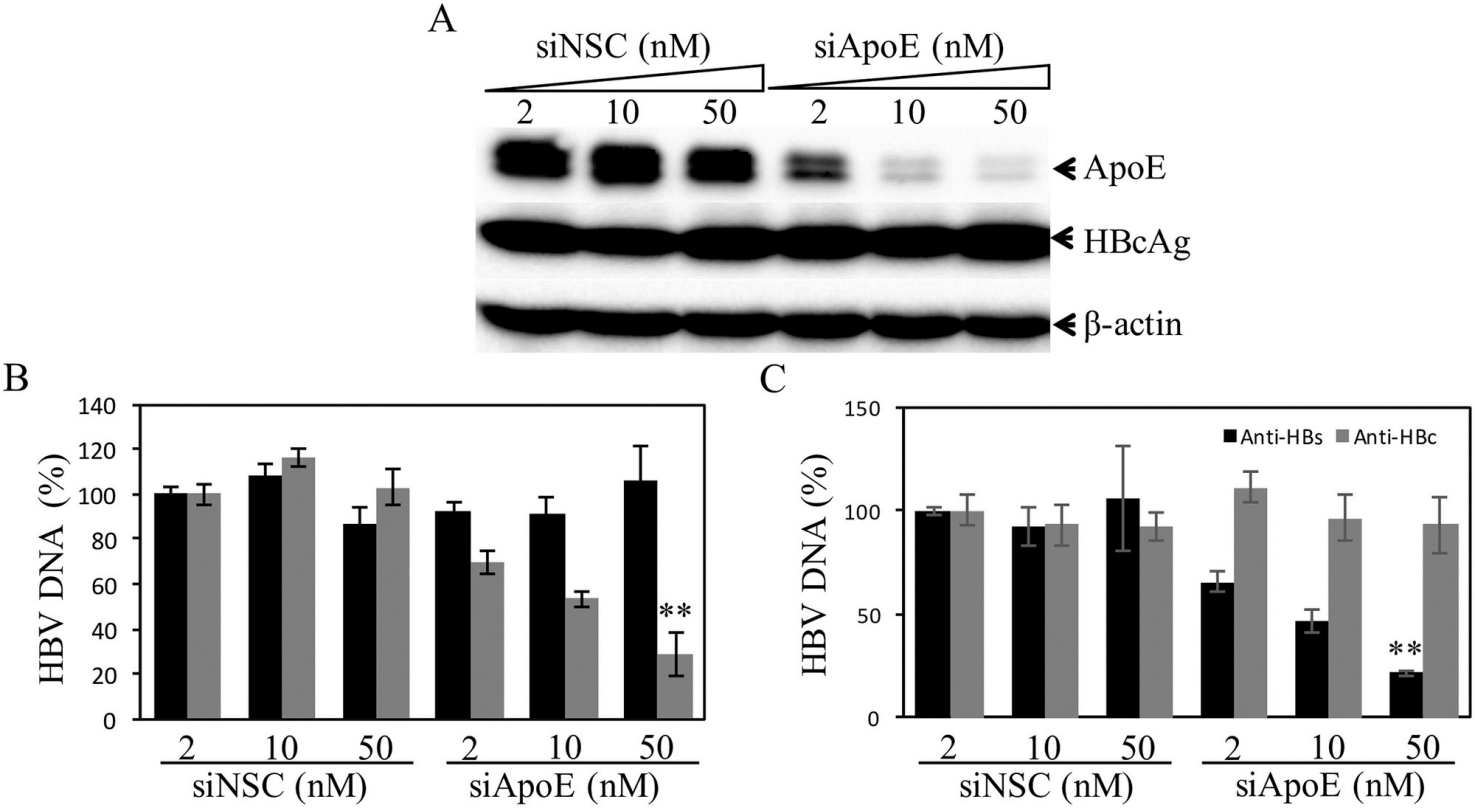
Reduction of HBV production by silencing apoE expression. The HBV-producing cell line HepAD38 in 24-well cell culture plates were transfected with apoE-specific siRNAs (siApoE) or a NSC siRNA (siNSC) at varying concentrations (0, 2, 10, and 50 nM). At 48 h p.t., siRNA-transfected cells were incubated with DME/F12 medium containing 4% FBS, 1% DMSO, and 5 μg/mL hydrocortisone. After 3-days culturing, cell lysates were collected for detection of apoE and HBc by WB (**A**). HBV DNA in the cell (black solid bar) and cell culture supernatants (grey solid bar) was extracted and quantified by qPCR (**B**). The cell culture supernatants were also used for IP of HBV virions and nonenveloped capsids using anti-HBs (solid black bars) and anti-HBc (solid grey bars) antibodies. HBV DNA extracted from immunoprecipitated virions and nucleocapsids was quantified by qPCR (**C**).

**Fig 8:**
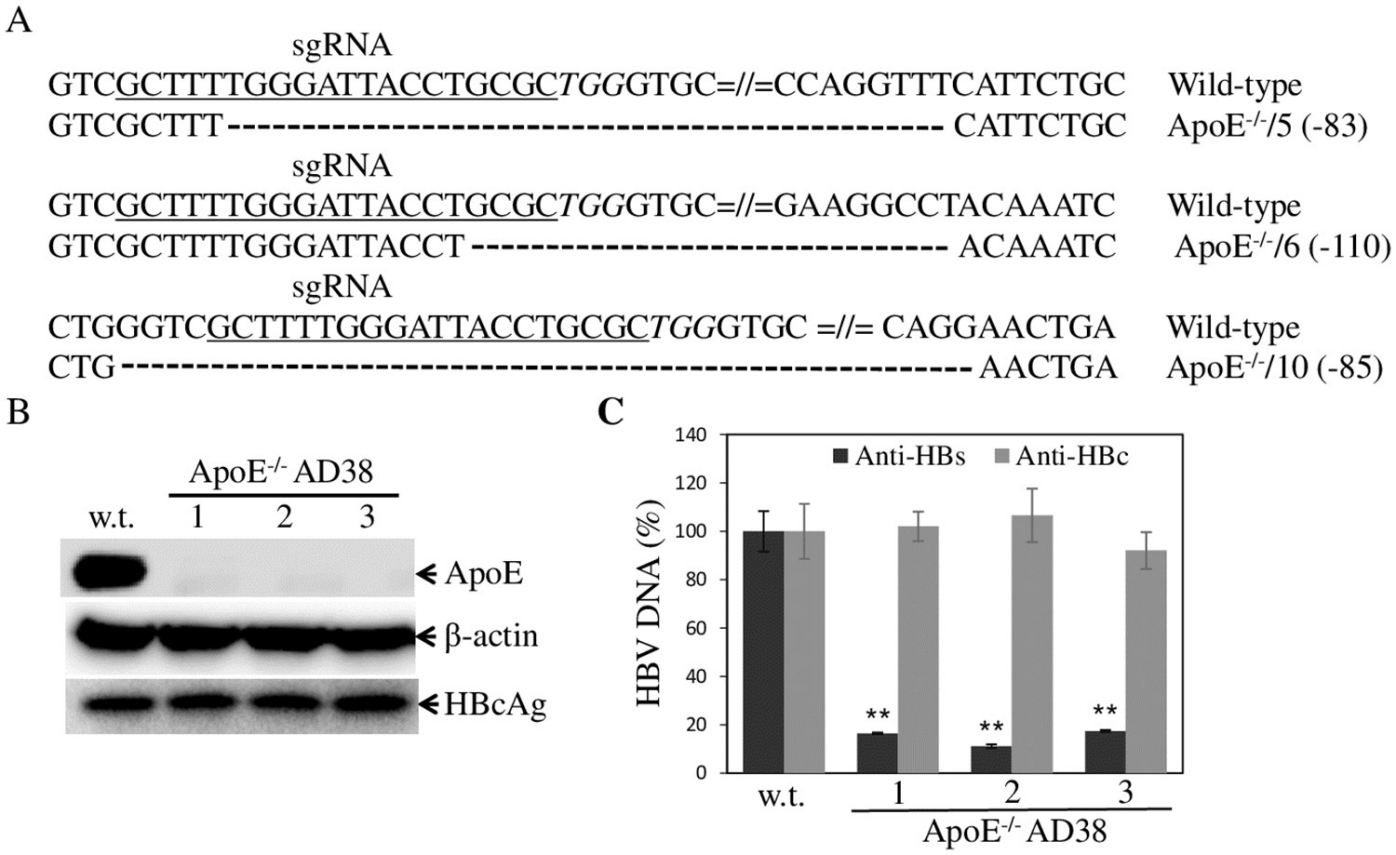
Effects of apoE gene knockout on HBV production. The construction of stable apoE gene knockout HepAD38 cell lines was the same as that in HepG2^NTCP^ (Fig 6). **A**. Confirmation of the apoE^-/-^ HepAD38 cell lines by DNA sequence analysis. Three apoE^-/-^ HepAD38 cell lines contained 83-nt, 85-nt, and 110-nt deletions, respectively, in the apoE ORF. **B**. Detection of apoE and HBc by WB in the apoE^-/-^ HepAd38 cell lines. **C**. Reduction of HBV production by apoE gene knockout. The parental (w.t.) and apoE^-/-^ HepAD38 cells in 24-well cell culture plates were cultured with DME/F12 medium containing 4% FBS, 1% DMSO, and 5 μg/mL hydrocortisone for 3 days. HBV virions and nonenveloped capsids were pulled down by IP using anti-HBs (solid black bars) and anti-HBc (solid grey bars) antibodies, respectively. Resulting HBV virions and nonenveloped capsids were used for extraction of HBV DNA, which was quantified by qPCR. Average values from three repeats were calculated and plotted in C. ** P<0.01.

We and others have previously shown that apoE is critical for HCV assembly and production besides its importance in HCV infection [41, 43, 53, 56, 57]. To determine if apoE affects HBV production, its expression in the HBV-producing cell line HepAD38 was silenced by apoE-specific siRNAs. As expected, apoE siRNAs efficiently silenced apoE expression in a dose-dependent manner. In contrast, the levels of HBcAg (Fig 7A) and HBV DNA (solid black bars, Fig 7B) in the HBV-producing cells remained unchanged, suggesting that apoE did not affect HBV DNA replication in the cell. However, the levels of HBV DNA in the cell culture supernatants were lowered by 30%, 45%, and 70% at apoE siRNA concentrations of 2, 10, and 50 nM, respectively (solid gray bars, Fig 7B). It has been previously shown that the HBV-infected or HBV-producing hepatocytes *in vitro* and *in vivo* also produced nonenveloped nucleocapsids (naked capsids) [58–61], which might confound the effect of apoE silencing on HBV production. To dissect the effects of apoE silencing on HBV and capsid production, HBV virions and nonenveloped capsids were pulled down by IP using anti-HBs and anti-HBc antibodies, respectively. Interestingly, the silence of apoE expression in HepAD38 cells did not affect the secretion of nonenveloped capsids as suggested by similar levels of HBV DNA extracted from anti-HBc-pulled down capsids (solid grey bars, Fig 7C). The levels of HBV DNA extracted from the anti-HBs-precipitated HBV were proportionally decreased by up to 80% at 50 nM concentration of apoE siRNAs (solid black bars, Fig 7C). These results suggest that apoE is required for efficient production of HBV virions but not nonenveloped capsids. To further confirm the importance of apoE for HBV production, apoE gene was subjected to knockout in the HBV-producing HepAD38 cells similar to the above-described apoE^-/-^ HepG2^NTCP^ cells (Fig 6). A total of three apoE^-/-^ HepAD38 cell lines were validated by DNA sequence analysis and the absence of apoE expression as determined by WB, including 83-nt, 85-nt, and 110-nt deletions in the apoE ORF (Fig 8A and 8B). The production of HBV virions and nonenveloped capsids in the supernatants was determined by qPCR quantification of HBV DNA extracted from HBV and nonenveloped capsid, which were pulled down by IP using anti-HBs and anti-HBc antibodies, respectively. HBV production was lowered by 80–90% in the apoE^-/-^ HepAD38 cells compared to that in the parental cells (solid black bars, Fig 8C). However, the levels of HBV DNA extracted from nonenveloped capsids remain unchanged (solid grey bars, Fig 8C). Taken together, these findings demonstrate that apoE is also critical for envelopment and/or production of HBV virions but does not play a role in the secretion of nonenveloped HBV capsids.

### Restoration of defective HBV infection and production by ectopic apoE expression

**Fig 9:**
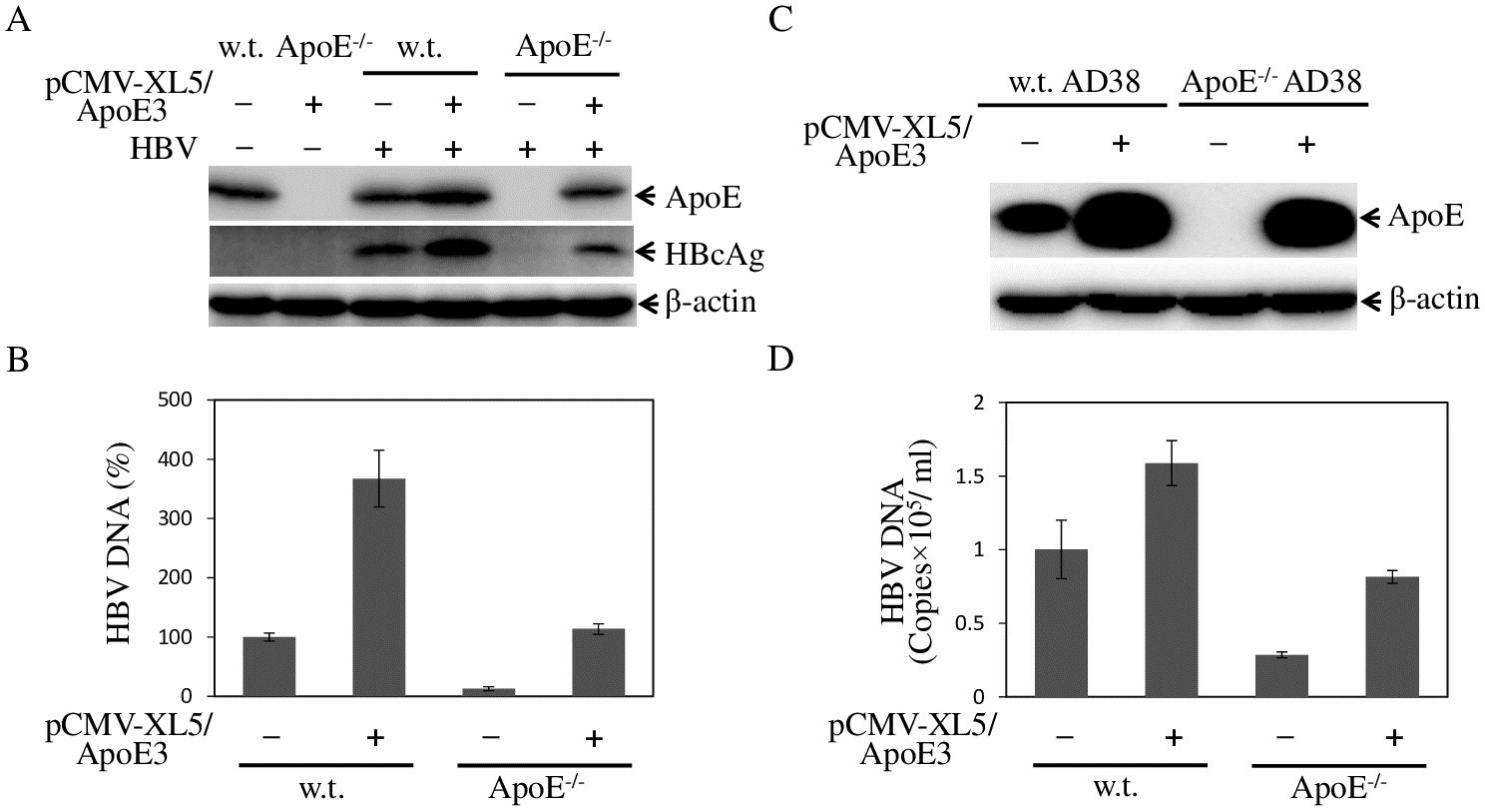
Restoration of defective HBV infection and production by ectopic apoE expression. ApoE^-/-^ HepG2^NTCP^ cells in 24-well cell culture plates were transfected with 1 μg pCMV-XL5-apoE3 or the vector DNA. At 48-h p.t., cells were infected with HBV at 37°C for 12 h. Upon removal of unbound HBV, cells were cultured with DME/F12 medium containing 4% FBS, 1% DMSO, and 5 μg/mL hydrocortisone for 4 days. The levels of apoE and HBc in the HBV-infected cells were determined by WB (**A**), whereas the levels of HBV DNA in the cell culture supernatants were quantified by qPCR (**B**). For ectopic apoE expression in the apoE^-/-^ HepAD38 cells, 1 μg of pCMV-XL5-apoE3 DNA or vector DNA was transfected into the parental (w.t. AD38) or apoE^-/-^ AD38 cells in 24-well cell culture plates. Cell culture medium was changed to DME/F12 with 4% FBS, 1% DMSO, and 5μg/ml hydrocortisone. After additional 3-days incubation, cell lysates were collected for detection of apoE and HBc by WB (**C**). HBV DNA in the cell culture supernatants was extracted and quantified by qPCR (**D**). The percentage of relative HBV DNA levels in B and D was based on average values of three independent experiments considering 100% of HBV DNA level in the parental HepG2 cells without ectopic apoE expression.

The defective HBV infection and production in the apoE^-/-^ HepG2^NTCP^ and HepAD38 cells could be caused by potential off-target effects associated with CRISPR/Cas9 gene-editing system. To exclude this possibility, we sought to determine whether the defect of HBV infection and production could be restored by ectopic expression of apoE. The apoE^-/-^ HepG2^NTCP^ cells were transfected with the apoE3-expressing vector pCMV-XL5-hApoE3. At 48-h p.t., cells were infected with HBV, followed by the detection of apoE and HBcAg. The cell culture supernatants were collected for quantification of HBV DNA. Interestingly, over-expression of apoE3 in parental HepG2^NTCP^ cells enhanced HBV infection by greater than 300%. More significantly, ectopic expression of apoE3 fully restored HBV infection in the apoE^-/-^ HepG2^NTCP^ cells (Fig 9A and 9B). Similarly, over-expression of apoE in parental HepAD38 cells further increased HBV production by about 60%. Ectopic expression of apoE3 in the apoE^-/-^ HepAD38 cells restored HBV production to 85% of that in parental HepAD38 cells (Fig 9C and 9D). Collectively, these results demonstrate that apoE is specifically required for efficient HBV infection and production.

## Discussion

In the present study, we have obtained substantial evidence demonstrating that human apoE is associated with infectious HBV and plays an important role in HBV infection. ApoE was found to be enriched in purified HBV, as demonstrated by its co-existence with HBcAg, LHBsAg, and HBV DNA in the same fractions (Fig 1A, 1B and 1D). There was a close correlation between apoE and HBV infection as shown by the peak level of HBV infectivity in fractions 6 and 7 (Fig 1C), which also contained the highest levels of apoE (Fig 1A). The association of apoE with HBV was further supported by a specific capture of HBV with an apoE monoclonal antibody similar to anti-HBs. Most of purified HBV could be specifically pulled down by anti-apoE and anti-HBs, as determined by the levels of HBV DNA and HBcAg in the immunoprecipitated virus (Fig 2A). Interestingly, the HBV-associated apoE was sensitive to trypsin digestion unlike HBcAg which only became sensitive to trypsin digestion upon disruption of the viral envelope by treatment with triton X-100 (Fig 2B). These findings suggest that apoE is likely exposed on the HBV envelope and may play a role in HBV infection. Indeed, HBV infectivity in PHHs and HepG2^NTCP^ could be efficiently neutralized by an apoE monoclonal antibody, which was added during HBV infection or during and after HBV infection, resulting in a greater than 90% reduction of HBcAg and HBV cccDNA in the cell and HBeAg and HBV DNA in the cell culture supernatants (Fig 3). However, HBV infection was not significantly affected when the apoE-blocking antibody was added to cell culture media after HBV infection (Fig 3A). These findings demonstrate that apoE on the viral envelope does play a critical role in HBV infection. Apart from the importance of the virus-associated apoE in HBV infection, apoE expression from target cells is also required for efficient HBV infection. Both silencing of apoE expression and knockout of apoE gene greatly decreased the permissiveness of HepG2^NTCP^ cells to HBV infection (Figs 4 and 5). However, silencing of apoB expression did not affect HBV infection (Fig 5), suggesting a specific requirement of apoE for HBV infection. We have not examined the effect of apoE silencing or knockout on HBV infection in PHH or other human hepatocyte cell lines besides HepG2^NTCP^ cell lines available in the lab. More importantly, the defect of HBV infection in the apoE^-/-^ HepG2^NTCP^ cells could be completely restored by ectopic expression of apoE (Fig 9A and 9B). However, HBV protein expression and DNA replication were not significantly affected in apoE knockdown or knockout HepAD38 cells (Figs 7 and 8), demonstrating that apoE does not play a role in HBV protein expression or DNA replication. These findings are similar to those observed for HCV. ApoE gene knockout was also found to greatly impair the susceptibility of Huh7.5 cells to HCV infection but not replication [54, 55]. Thus, it is likely that apoE may mediate HBV infection via a similar mechanism to HCV infection, which is warranted for future investigation.

The underlying molecular mechanism for the apoE-mediated promotion of HBV infection is not clear, particularly for apoE expressed in target cells. In an analogy to its role in HCV infection [45, 47, 62], apoE may also mediate HBV cell attachment. This possibility is supported by circumstantial evidence derived from previous studies by others. Earlier studies found that heparin was able to block HBV infection in cell culture. Similarly, cells treated with heparinase became less susceptible to HBV infection, suggesting a role of HSPG in the apoE-mediated HBV cell attachment [63, 64]. In fact, one of the HSPG core proteins, glypican 5 (GPC5), was found as an HBV entry factor [65]. Taken together, these earlier findings suggest that HSPGs serve as HBV attachment receptors. HSPGs are known to be apoE-binding receptors and play a pivot role in the metabolism of apoE-containing lipoproteins [66–68]. Our previous studies demonstrated that apoE mediates HCV cell attachment through interaction with HSPGs [44, 45, 47, 48]. However, there is no good assay to confirm the role of apoE in HBV cell attachment unlike HCV. Neither is clear how apoE expressed in target cells facilitate HBV and HCV infection. In the case of HCV, several studies by others suggested that apoE binds HCV envelope protein E1 and E2 [49, 69]. The interaction between E1/E2 and apoE may stabilize HCV attachment mediated through the interaction between apoE and HSPGs. Similarly, our preliminary results appeared to suggest an interaction between apoE and the preS1 domain of LHBsAg. The interaction between apoE and preS1 will further promote HBV attachment mediated through the binding of apoE to the cell surface HSPGs. Alternatively, transfer of the exchangeable apoE from target cells to virions during HBV infection could also enhance HBV infection, as demonstrated by extracellular apoE-mediated promotion of HCV infection [54, 55].

Besides its importance in HBV infection, apoE is also critical for efficient HBV production similar to its role in HCV assembly, maturation, and release [41, 43, 49, 53, 56, 57]. Silencing of apoE expression in the HBV-producing HepAD38 cells significantly reduced HBV production in proportion to the siRNA-induced down-regulation of apoE expression (Fig 7). Likewise, apoE gene knockout decreased HBV production by 90%, as determined by the levels of HBV DNA extracted from anti-HBs-precipitated HBV virions in the cell culture supernatants among three different HepAD38 cell lines (Fig 8). However, the levels of HBcAg and HBV DNA in the apoE-silencing (Fig 7) or knockout (Fig 8) HepAD38 cells remained the same, demonstrating that apoE does not play a significant role in HBV protein expression or DNA replication. Neither was the secretion of nonenveloped capsids affected by silencing of apoE expression (Fig 7C) and knockout of apoE gene (Fig 8C) in the HBV-producing HepAD38 cells. Whether apoE play any role in the secretion of HBV subviral particles remains unknown. We used anti-HBs to pull down HBV virions and subviral particles but could not discriminate them by an immunoblot assay with a preS1-specific monoclonal antibody upon density gradient ultracentrifugation and fractionation (Fig 1D). Nevertheless, we speculate that apoE may play a role in HBV post-envelopment during virion assembly and maturation steps similar to its function in HCV morphogenesis [52]. We and others had previously found that apoE is required for the formation of infectious HCV particles through a specific interaction with NS5A [41, 43]. Mutations introduced to apoE and NS5A that disrupted the apoE-NS5A interaction resulted in an impairment of HCV production [41, 43]. The studies by others suggested an interaction of apoE with HCV envelope proteins E1/E2 [49, 69]. It is possible that the interaction between apoE and preS1 domain of the LHBsAg might promote HBV envelopment and/or egress through an unknown mechanism. Whether HBV morphogenesis and egress depend on the lipoprotein secretory pathway remains unknown. The role of VLDL secretion pathway in HCV morphogenesis and production has been controversial [46, 70, 71]. The siRNA-mediated knockdown of apoB expression and specific inhibitors of microsomal triglyceride transfer protein (MTTP), which is essential for assembly and secretion of VLDL [72, 73], did not affect HCV production, as demonstrated by our previous study [46]. It will be interesting to determine if lipoproteins of different densities play any role in HBV infection and production *in vivo*. Whether apoE isoforms (E2, E3, and E4) exhibit difference in HBV infection and production is warranted for further investigation. Nevertheless, it remains enigmatic how apoE promotes HBV production. The identification of the cellular gene(s) and/or pathway responsible for the incorporation of apoE into HBV particles may provide novel molecular target(s) for intervention of HBV infection.

The close association of HBV with apoE or apoE-containing lipoproteins may be not only important for HBV infection and production but also viral pathogenesis by evading host immune response [74]. It was previously shown that apoE incorporated into HCV could interfere with the activity of HCV-neutralizing monoclonal antibodies in cell culture, suggesting a role of apoE in persistent HCV infection *in vivo* [75, 76]. It is conceivable that the HBV-associated apoE may also play a critical role in chronic HBV infection by blocking the accessibility of HBV-neutralizing antibodies and evading cellular immune response targeting HBV-infected cells. Inhibitors that interfere with apoE biogenesis, secretion, and its interaction with cell surface receptors may serve as potential anti-HBV agents. It will be interesting to determine whether the levels of apoE and/or apoE-containing lipoproteins in the plasma of hepatitis B patients correlate with the viral load and/or the outcomes of HBV infection.

## Materials and methods

### Cells and virus

A puromycin-resistant HepG2^NTCP^ cell line (HepG2^NTCP^-P3) was described in our previous work [25]. The HBV-producing cell line HepAD38 was described previously [77]. Both HepG2^NTCP^ and HepAD38 were grown in DME/F12 medium containing 10% of fetal bovine serum (FBS, Atlanta Biologicals). Primary human hepatocytes (PHHs, lot#4405C) were purchased from Lonza and were cultured in Power Primary HEP medium (Takara, San Francisco, CA). HEK293T cells were from ATCC and grown in DMEM containing 10% of FBS as previously reported [78]. Cell culture flasks and plates were coated with 50 μg/mL of rat tail collagen type I (Corning). HBV obtained from HepAD38 cells were concentrated by precipitation with 10% polyethylene glycol (PEG) 8,000 (Hampton Research). The genome copy numbers of HBV DNA were quantified by a real-time PCR method.

### Antibodies and reagents

ApoE-specific monoclonal antibodies 23 (mAb23) and WuE4 (ATCC) were described in our previous work [26, 45]. ApoE mAb23 was purified by GenScript (Piscataway, NJ). HBV core-specific monoclonal antibody (T2221) was from Tokyo Future Style [25]. HBV preS1 antibody (SC57761) and normal mouse and rabbit IgGs were purchased from Santa Cruz Biotechnology. Human β-actin monoclonal antibody (AC15) and protein G agarose were obtained from Sigma-Aldrich. HRP-conjugated goat anti-mouse antibody was from Cell Signaling. Anti-HBs antibody (hepatitis B immune globulin, HBIG) was from Nanyue Biopharmaceuticals Inc, China. Clarity Max Western blotting ECL substrate was purchased from Bio-Rad. ApoB- and ApoE-specific Smartpool siRNAs and a nonspecific control (NSC) siRNA were from Dharmacon [26]. One-Step qRT-PCR kits were from Thermo Scientific. Taq 5× master mix was from New England Biolab. HBeAg chemiluminescence immunoassay (CLIA) kits were purchased from Autobio Diagnostics Co. (Zhengzhou, China) as previously described [19, 25]. Co-IP kits were purchased from Pierce (Thermo-Fisher). Protein A magnetic beads (Dynabeads) were from Thermo Fisher. TRIzol reagent was from Invitrogen. Genome DNA isolation kit was from QIAGEN. Exonucleases ((Exo I) and III (Exo III) were purchased from New England Biolabs.

### HBV purification and characterization

HBV grown from HepAD38 cells was concentrated by precipitation with 10% polyethylene glycol 8000 (PEG 8000). Concentrated HBV was used for IP of HBV and subviral particles using anti-HBs antibodies. A total of 100 μg of anti-HBs was incubated with 50 mg of protein A magnetic Dynabeads in 500 μl volume at 4°C overnight. Upon extensive washing, anti-HBs-conjugated beads were mixed with 1 ml of PEG-concentrated HBV with rotation at 4°C overnight, followed by washing with PBS three times. HBV virions and subviral particles pulled down by IP were released into 500 μl of glycine (50 mM) buffer at room temperature for 10 minutes, followed by neutralization with the addition of 500 μl of Tris-HCl (pH7.5). Immunoprecipitated HBV or PEG-concentrated virus was subjected to purification by Cesium chloride (CsCl) gradient ultracentrifugation. Briefly, equal volume of 2 X CsCl solutions (49.74g CsCl dissolved to a total of 100 ml Tris-Sodium Chloride-EDTA with 0.05% β-Mercaptoethanol) was mixed with equal volume of concentrated HBV, followed by centrifugation in a Beckman SW55Ti rotor at 50,000 rpm at 4°C for 94 h. The gradient was fractionated from bottom to top. HBcAg, LHBsAg, and apoE in each fraction were detected by Western blot (WB). HBV DNA in each fraction was extracted with a QIAGEN viral DNA isolation kit and was quantified by a real-time PCR method. HBV infectivity in each fraction was determined by an HBV infection assay using HepG2^NTCP^-P3 cells in 24-well cell culture plates. Each fraction of 25 μL was diluted with DME/F12 medium into a 300 μL volume containing 4% PEG-8000 and was used to infect HepG2^NTCP^-P3 cells at 37°C for 12 hours. At 4 days post-infection (p.i.), the HBV-infected HepG2^NTCP^-P3 cells were lysed with a RIPA buffer. Cell lysates were used for quantification of HBcAg by WB using the HBc monoclonal antibody T2221 [25].

### Trypsin digestion of purified HBV

Purified HBV particles were treated with trypsin in the absence or presence of 1% Triton X-100 for 1h at 37°C, as described previously [46]. Trypsin reactions were terminated by the addition of 1×SDS protein loading buffer. After trypsin digestion, HBcAg and apoE were determined by WB using specific monoclonal antibodies.

### HBV pull-down assay

A total of 50 μg of purified apoE mAb23, anti-HBs, or a normal mouse IgG (nmIgG) was coupled to protein G agarose at room temperature for 2 hours. The antibody-conjugated agarose was then mixed with 10 μL of purified HBV (Fraction 7) at 4°C overnight, similar to HCV IP [46]. After extensive washing, the precipitated HBV was used for detection of HBcAg or extraction of HBV DNA, which was subsequently quantified by qPCR using HBV sequence-specific primers and probe as described previously [13, 15, 25]. For pull-down of HBV virions and subviral particles as well as nonenveloped capsids by IP, 5 mg of protein A magnetic beads (Dynabeads) were conjugated with 10 μg of anti-HBs and anti-HBc, respectively, and then incubated with 250 μl of cell culture supernatants from apoE-silencing or apoE^-/-^ HepAD38 cells. Upon washing with ice-cold PBS, HBV virions and naked capsids were eluted out by incubation with 100 μl of 50 mM glycine buffer, followed by neutralization with 100 μl of 1M Tris buffer (pH7.5). HBV DNA was extracted using QIAGEN genome DNA extraction kit.

### HBV infection assay

HepG2^NTCP^-P3 or PHH cells were seeded into 24-well cell culture plates at a density 1×10^5^ per well at one day prior to infection. HepG2^NTCP^-P3 cells were infected with HBV at a multiplicity of infection (m.o.i.) of about 100 copies of genome equivalent in the present of 4% PEG 8000 for 12 hours except otherwise indicated. HBV-infected cells were cultured in DME/F12 medium containing 4% FBS, 1% DMSO, and 5 μg/mL hydrocortisone for 4 days or otherwise as indicated.

### DNA extraction

HBV DNA in the cell culture supernatants was extracted with QIAGEN DNA isolation kits. HBV cccDNA in the cell was isolated using the Hirt method [79], followed by treatment with exonucleases I (Exo I) and III (Exo III), which removes DNAs with open 5’ and 3’ ends, including HBV rcDNA [13, 25, 80].

### Quantification of HBeAg by chemiluminescence immunoassay

Chemiluminescence immunoassay kits purchased from Autobio Diagnostics Co. (Zhengzhou, China) were used to quantify the levels of HBeAg in the cell culture supernatants as described previously [19, 25].

### Western blot analysis

Bio-Rad protein assay dye was used to measure protein concentration of cell lysates. A total of 25 μg protein from each sample was separated by electrophoresis in SDS-PAGE and then transferred onto a polyvinylidene difluoride (PVDF) membrane using a semidry blotter (Bio-Rad). Immunoblot analysis was done using Clarity Max western ECL substrate (Bio-Rad) and mouse monoclonal antibodies specific to HBcAg, LHBsAg, apoB, apoE, or β-actin.

### HBV neutralization by an apoE-blocking monoclonal antibody

For neutralization of HBV infectivity during HBV infection, concentrated HBV in 4% PEG was used to dilute the apoE-blocking monoclonal antibody 23 (mAb23) into varying concentrations (0, 0.4, 2, and 10 μg/mL). HBV in the presence or absence of the apoE mAb23 was subsequently used to infect HepG2^NTCP^-P3 or PHH cells in 24-well cell culture plates. A normal mouse IgG was used as a negative control. After 12-h incubation at 37°C, the HBV-infected cells were cultured in DME/F12 medium containing 4% FBS, 1%DMSO, 5 μg/μL hydrocortisone. Additionally, apoE mAb23 was added to cell culture media after the above HBV infection in the presence of apoE mAb23, referring as mAb23 presence during and after HBV infection. ApoE mAb23 was also tested for its inhibitory effect on HBV infection when added to cell culture media only after HBV infection. At 4-days post-infection, the levels of HBcAg in the infected cells were determined by WB. HBV cccDNA in the cell was extracted with the Hirt method. The levels of HBeAg and HBV DNA in the supernatants were quantified by chemiluminescence immunoassay and qPCR, respectively.

### siRNA-mediated silencing of apoE or apoB expression

The apoE-, apoB-specific siRNA and a NSC siRNA were described previously [26, 46]. HepG2^NTCP^-P3 cells in 24-well cell culture plates were transfected with increasing concentrations (0, 2, 10 and 50 nM) of apoE, apoB or NSC siRNA using RNAiMax reagent (Invitrogen). At 48 h post-transfection (p.t.), HepG2^NTCP^-P3 cells were infected with HBV in 4% PEG at a m.o.i. of about 100 copies of genome equivalent for 12 h at 37°C. After an additional 4-days incubation, cells were lysed for detection of apoE and HBcAg by WB, whereas the media were collected for quantifying the levels of HBV DNA and HBeAg by quantitative PCR (qPCR), and chemiluminescence immunoassay, respectively.

### CRISPR/Cas9-mediated apoE gene knockout in HepAD38 and HepG2^NTCP^

The lentiCRISPRv2-blasticidin vector previously made in the lab was digested with BsmBI. The apoE gene-specific sgRNA was designed based on the website http://crispr.mit.edu (Feng Zhang). The apoE-specific sgRNA cDNA was constructed by annealing two synthetic oligonucleotides, apoE/F (5’-CACCGGCTTTTGGGATTACCTGCGC-3’) and apoE/R (5’-AAACCGCGCAGGTAATCCCAAAAGCC-3’). The double-stranded DNA fragment was inserted into the BsmBI-digested lentiCRISPRv2-blasticidin vector in the same way as described previously [44]. Resulting plasmid DNA construct was confirmed by DNA sequence analysis and was designated pLentiCRISPRv2-Blast/ApoE-sgRNA. For lentiviral production, HEK293T cells in 6-well plates were transfected with pVSVg, psPAX2, and pLentiCRISPRv2-Blast/ApoE-sgRNA using lipofectamine 3000 reagent according to the manufacturer’s instructions (Invitrogen). At 72 h p.t., the supernatant was collected and filtered through a 0.45-m low-protein-binding membrane unit (Millipore). Resulting lentivirus expressing apoE sgRNA and CRISPR/Cas9 was used to transduce HepAD38 and HepG2^NTCP^ cells. Stable apoE-knockout cell lines were selected with 5 μg/mL blasticidin and were confirmed by WB and DNA sequence analyses.

### Restoration of HBV infection and production by ectopic apoE expression

An apoE expression plasmid pCMV-XL5-apoE3 was made previously [43]. The parental and apoE^-/-^ HepG2^NTCP^ cells were seeded at 1 × 10^5^ cells/well in 24-well cell culture plates. A total of 1 μg of pCMV-XL5-apoE3 was transfected into HepG2^NTCP^cells using Lipofectamine 3000 reagent. At 48 h p.t., the DNA-transfected cells were infected with HBV in 4% PEG 8000 at about 100 copies of genome equivalent for 12 h at 37°C. After incubation for 4 days, cells were lysed for measuring apoE and HBc by WB, whereas the cell culture supernatants were collected for quantifying the levels of HBV DNA by qPCR, respectively.

### Real time PCR and RT-PCR

HBV cccDNA was extracted by the Hirt method [79]. HBV DNA was quantified by a real-time PCR method using two HBV-specific primers: 5′-GAGTGTGGATTCGCACTCC-3′ (forward) and 5′-GAGG CGAGGGAGTTCTTCT-3′ (reverse). HBV cccDNA was quantified using primers 5′- TCATCTGCCGGACCGTGTGC-3′ (forward) and 5′- TCCCGATACAGAGC TGAGGCGG-3′ (reverse) and probe HBV-cccP: 5’-FAM-TTCAAGCCTCCAAG CTGTGCC TTGGGTGG C-TAMRA -3’. Internal control Mitochondrial DNA was quantified using primer mitoF: 5’-CCCTCTCGGCCCTCCTAATAACCT-3′ (forward) and mitoR: 5’-GCCTTCTCGTATAACATC GCGTCA-3’ (reverse). The qPCR was carried out with TaqMan SYBR Green Master Mix (Applied Biosystems) or iTaq Universal Probes Supermix (Bio-Rad) and at 95 °C for 10 min (1 cycle), and 95 °C for 15 s and 60 °C for 60 s (40 cycles).

### Statistical analysis

Statistical analyses were conducted using Prism5 software (Graphpad Software). Results are shown as means ± standard deviations (SD) of the data obtained from three independent experiments or triplicates as indicated. Comparisons between samples were done using the paired two-tailed t test. P values of < 0.01 were considered statistically very significant.
